# Antibacterial effect of an herbal toothpaste containing Bamboo salt: a randomized double-blinded controlled clinical trial

**DOI:** 10.1186/s12903-022-02224-z

**Published:** 2022-05-19

**Authors:** Mina Biria, Yasaman Rezvani, Romina roodgarian, Abbas Rabbani, Parastoo Iranparvar

**Affiliations:** 1 Department of Pediatric Dentistry, School of Dentistry, Shahid Beheshti University of Medical Sciences, Tehran, Iran; 2 Dental Research Center, School of Dentistry, Shahid Beheshti University of Medical Sciences, Tehran, Iran

**Keywords:** Toothpastes, *Streptococcus mutans*, *Lactobacillus*, Bamboo salt, Integrative medicine

## Abstract

**Background:**

The inclusion of herbal antibacterial agents in the composition of toothpastes is becoming increasingly popular, due to lower side effects. The present study intended to investigate the antibacterial efficacy of a herbal toothpaste containing Bamboo salt on cariogenic oral bacteria.

**Methods:**

The present double-blinded parallel randomized controlled clinical trial was conducted on 60 dental students (age range: 18–30). Following the baseline saliva sampling, the participants were randomly assigned into the case and control groups, to use the Bamboo salt herbal toothpaste and conventional non-herbal toothpaste, respectively. They were instructed to brush their teeth twice a day using the Bass technique. Saliva sampling was repeated after four weeks. The salivary counts of *Streptococcus mutans* and *Lactobacillus* at baseline and 4-week follow-up were determined and presented as the logarithm of colony-forming units per milliliter (log CFU/mL).

**Results:**

A significant decrease in salivary *Streptococcus mutans* and *Lactobacillus* was observed using both toothpastes (*P < 0.001). The difference between the antibacterial efficacy of two toothpaste types on *Streptococcus mutans* and *Lactobacillus* was not statistically significant (*P* = 0.530, and *P* = 0.137, respectively).

**Conclusion:**

Due to the comparable efficacy of the investigated herbal toothpaste with conventional toothpaste, it potentially qualifies as a complementary agent for self-care oral hygiene procedures.

*Trial registration*: This trial was registered in the “Iranian Registry of Clinical Trials” (IRCT20210414050964N1) on 21/06/2021.

**Supplementary Information:**

The online version contains supplementary material available at 10.1186/s12903-022-02224-z.

## Background

Dental caries and periodontal problems are widespread oral bacterial diseases [1]. There is a rich ecosystem in the oral cavity, with a countless number of microorganisms [2]. Despite the multifactorial nature of both periodontal diseases and dental caries, it is dental plaque bacteria that plays a key role in their onset and progression [3].* Streptococcus mutans* is known as the main initiating pathogen of dental caries, colonizing on tooth surfaces by several mechanisms [4]. In the presence of surface-adsorbed salivary α-amylase, carbohydrates are degraded by bacterial enzymes, resulting in organic acids which demineralize enamel [5].* Lactobacilli* are Gram-positive bacteria that are transmitted to the oral cavity during the first few years of life [6]. *Lactobacilli* are considered a major contributor to the progression of caries [7]. Increased levels of *Streptococcus mutans* and *Lactobacillus* in the oral microbiota is associated with the onset and progression of tooth demineralization and caries [8, 9].

Co-application of brushing with toothpaste containing certain chemical agents helps remove pathogenic biofilm and reduces bacterial repopulation on the enamel. Toothbrushing is associated with a decrease in oral bacterial levels and maintenance of a healthy balance of oral flora on tooth surfaces [10]. Typically, conventional toothpaste types contain triclosan and fluoride as main antibacterial ingredients. It has been proven that these ingredients have anti-cariogenic properties [11]. In recent years, there has been an increased interest in therapeutic herbal ingredients worldwide, due to reduced side effects and better availability, leading to the popularity of herbal toothpastes. With the increasing availability of herbal toothpaste brands on the market, the efficacy of these products on oral bacteria has been studied extensively in recent years [12–18].

Bamboo salt, which is a popular herbal ingredient in Korean folk medicine, has been shown to exhibit anti-inflammatory, antimicrobial, and antioxidant properties [19, 20]. Traditionally, Bamboo salt is made by packing sea salt in bamboo tubes, plugging the ends with mud, and baking it on pine firewood. This method results in higher mineral content of calcium, potassium, copper, and zinc ions in Bamboo salt compared to sun-dried salts while maintaining its main ingredient, i.e., sodium chloride [20, 21]. Mineral-rich Bamboo salt is highly alkaline (pH = 11.4), making for its potent antioxidant action [22].

Toothpaste containing Bamboo salt is proposed to reduce plaque and gingivitis, whiten teeth, decrease demineralization, decrease tooth hypersensitivity, and strengthen tooth enamel [23, 24]. The antimicrobial efficacy of Bamboo salt on *Streptococcus mutans* has recently been observed in an in-vitro study [21]; however, its in-vivo efficacy against cariogenic microorganisms remains unknown.

Considering the increasing popularity of herbal toothpaste types, and in order to provide evidence-based recommendations to the patients [18], the present randomized controlled clinical trial was designed to compare the antimicrobial efficacy of Tiger Herb® toothpaste (containing Bamboo Salt) with Crest Complete® toothpaste as the control, on *Streptococcus mutans* and *Lactobacillus*.

## Methods

### Participants

The present double-blinded parallel randomized controlled clinical trial was conducted at Shahid Beheshti School of Dentistry, after it was approved by the committee for ethics in research, school of dentistry, Shahid Beheshti University of Medical Sciences (IR.SBMU.DRC.REC.1399.033). The study design was in accordance with the Helsinki Declaration of Human Rights and was also registered in the “Iranian Registry of Clinical Trials” (IRCT20210414050964N1) on 21/06/2021. Each participant was included in the study after reading, understanding, and completing the written informed consent document.

200 dental students (18–30 years old) were screened by routine dental examination and finally, 60 participants were recruited for the study during a 2-week period. All participants had a history of routine daily toothbrushing with fluoride-containing toothpastes. The exclusion criteria included the following: having a periodontal pocket depth of more than three millimeters, receiving antibiotic or anti-inflammatory drugs during the past month prior to the study, history of systemic diseases, allergic reaction to the toothpaste, smoking, having orthodontic appliances, or presence of untreated dental caries.

### Sample size

The sample size was calculated based on a previous study to be 23 in each group, which was increased to 30 to improve the validity of the study and compensate for possible sample loss during the follow-up period [25]. The following formula was used to estimate the sample size:

$$\begin{aligned} & n = \frac{{(Z_{{1 - \propto/ 2}}+ Z_{1 - \beta } )^{2 } \left( {\sigma_{1}^{2} + \sigma_{2}^{2} } \right)}}{{(\mu_{1} - \mu_{2} )^{2} }} \\ & \left( {\varvec{\alpha}} = 0.05;\user2{ \beta } = 0.2 \to {\varvec{power}} = 80\user2{\% };\user2{ drop out} \right.\\ &\left.\quad= 20\user2{\% };\user2{ \mu }_{1} - {\varvec{\mu}}_{2} = 5.2;\user2{ \sigma }_{1} = 6.5;\user2{ \sigma }_{2} = 5.6 \right) \\ \end{aligned}$$

### Clinical procedure

The participants were coded from 01 to 60 and were randomly assigned into the control and case groups (allocation ratio = 1:1), to use the conventional non-herbal toothpaste (Crest® Complete, Procter and Gamble Plaza, Cincinnati, United States), and the Bamboo salt toothpaste (Tiger Herb®, LG, Korea), respectively. The composition of examined toothpaste types is listed in Table 1.

**Table 1:** List of ingredients in the two toothpaste types

Toothpaste type	Ingredients	Manufacturer
Tiger Herb®	Bamboo salt, Hydrated Silica, Dipotassium Glycyrrhizinate, Sodium Fluoride, Sorbitol Solution (70%), Cellulose Gum, Sodium Saccharin, Sodium Lauryl Sulfate, Sodium Bisulfate, Blue 1 (E142090), Yellow 10 (47,005) Flavor, Water	LG, Korea
Crest® Complete	Aqua, Sorbitol, Hydrated Silica, Sodium Lauryl Sulfate, Cellulose Gum, Aroma, Zinc Citrate, Chondrus Crispus Powder, Sodium Fluoride (1450 ppm), Sodium Saccharin, Hydroxyl Ethylcellulose, Cl 77,891, Sodium Citrate, Stannous Chloride, Silica, Glycerin and Cl 74,160	Procter and Gamble Plaza, United States

The information labels on all toothpaste tubes were removed and their packages were all designed similarly and coded as 1 or 2, to ensure the blinding of participants to the contents of toothpaste tubes. This was performed by a dental assistant who would not participate in the following random allocation process.

Randomization was performed using a random table number by Microsoft Excel (Microsoft®, Washington, US), and each participant received either tube 1 or 2, based on odd or even numbers, respectively. This allocation process was performed by a dental student (A.R.), who was blinded to the codes. The patient code and toothpaste code were recorded in a chart for further reference.

All participants were instructed to brush their teeth with a medium bristled brush (Oral B Pro Expert Brush, Procter & Gamble, United States) for two minutes using the Bass technique twice a day (after breakfast and before bedtime) for four weeks [26], using the designated toothpaste type. They were also instructed to use non-fluoridated dental floss (Oral B essential floss, Procter & Gamble, United States) every night before going to bed, and refrain from using any mouthwashes or other fluoride-containing products. In order to ensure the compliance of participants to follow the recommendations, weekly reminder phone calls were scheduled. In addition, the participants were asked to return their assigned tubes at the end of the study, in order to verify how much toothpaste was used.

### Bass technique

Bass technique is a brushing method recommended for routine patients with or without periodontal involvement. In this technique, the head of the brush is kept parallel to the occlusal plane, with the brush head covering almost 3–4 teeth starting from the distal most teeth of the arch. The bristles are placed at the gingival margin at an angle of 45 degrees to the long axis of the tooth. Gentle vibratory pressure is given using short back and forth movement dislodging the tips of the bristles. The Bass technique places emphasis on the removal of plaque from the area above and just below the gingival margin [27].

### Safety considerations

During the trial process, none of the participants were refrained from toothbrushing. The oral health of participants was assured at baseline examination and cases with active caries, periodontal problems, or allergies were excluded. The participants were contacted during the trial by weekly telephone calls, to ensure the absence of potential adverse effects. Since the trial was performed in a dental school and participants were dental students, all of them were assured to contact the pediatric dentistry department in case of any inconvenience caused. In case of any potential adverse reaction, such as allergy to toothpaste contents, supportive care would be provided immediately, and the participant excluded from the study. The participants were also free to stop participating in the trial whenever they wanted.

### Saliva sampling

Saliva sampling in both groups was performed at baseline and after four weeks of toothpaste use. At both time points, samples were collected before breakfast and one hour after toothbrushing with a medium-bristled brush (Oral B Pro Expert Brush, Procter & Gamble, United States) without any toothpaste, followed by drinking a cup of water. The participants were asked to sit for five minutes while the saliva was being accumulated in their oral cavity. Two milliliters of unstimulated resting saliva were then collected using disposable syringes (Ulticare insulin syringe, Ultimed, Minnesota, USA) and transferred into sterile microtubes (SPL life sciences, Korea). Thereafter, the microtubes were coded, stored in ice containers, and immediately transferred to the laboratory of microbiology.

### Microbiological studies

The saliva samples were diluted by 1/10 and then they were transferred to the Mitis Salivarius Agar (MSA) and MRS-Agar (MRS-A) containing plates (Merck KGaA, Darmstadt, Germany). All plates were then incubated at 37 ºC for 24–48 h (Memmert, Hong Kong) (Table 2). Colonies of *Streptococcus mutans* and *Lactobacillus* were counted after gram staining, using an automatic colony counter (Scan® 500, Interscience, Saint Nom, France) by two experienced microbiologists who were “blinded” to the administered toothpaste type. The inter-examiner reliability was determined using the Kappa agreement coefficient (k = 0.9).

**Table 2:** List of examined microorganisms and their culture media

Bacteria	Culture media	Manufacturer	Incubation
*S. mutans*^a^	Mitis Salivarius Agar (MSA)	Merck KGaA, Darmstadt, Germany	24 h, in CO_2_-containing incubator
*L. casei*^b^	MRS-Agar (MRS-A)	Merck KGaA, Darmstadt, Germany	48 h

^a^*S. Mutans* = *Streptococcus mutans*, ^b^*L. casei* = *Lactobacillus casei*

### Statistical analysis

Statistical analysis was performed using SPSS software version 25 (SPSS Inc., Chicago, IL, USA) (α = 0.05) (confidence interval = 95%). The logarithm of colony counts was calculated. Numerical data were presented as the mean and standard deviation (Mean ± SD). The normal distribution of data was assessed by the Kolmogorov–Smirnov test. Independent samples t-test and Paired samples t-test were used for inter-group and intra-group comparisons, respectively. The study was based on a per-protocol (PP) analysis since there were no planned or unplanned crossovers.

## Results

**Fig. 1:**
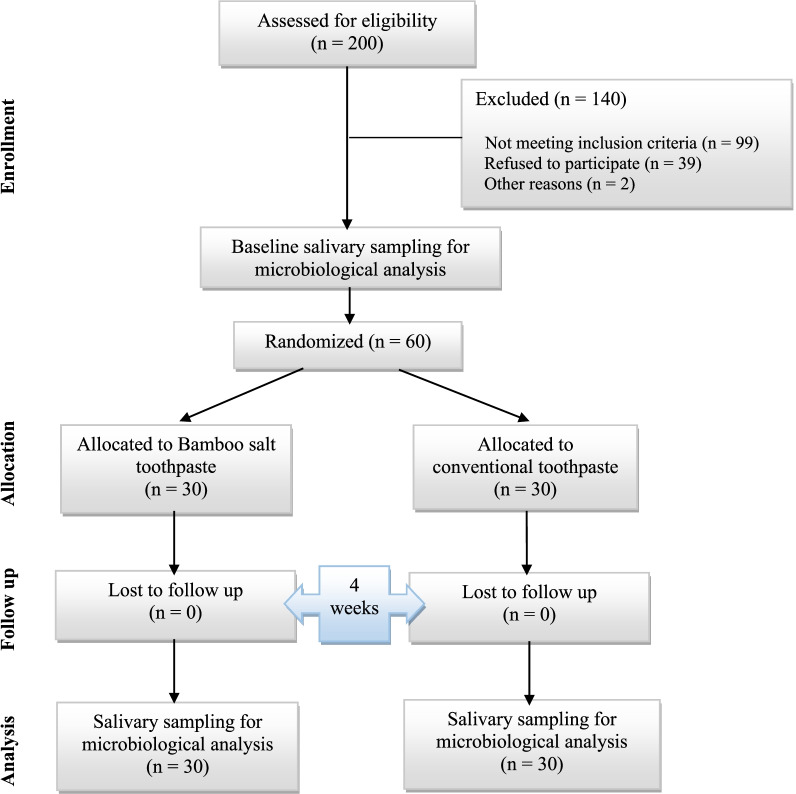
Flowchart of participants from baseline to 4-week follow up

Sixty participants with a mean age of 24.5 years were included in the study and allocated into two groups with a 1:1 ratio. The basic demographic characteristics of the participants among the two study groups are presented in Table 3. No sample loss occurred until the 4-week follow up session, although its possibility was anticipated in sample size calculation. The flow chart of participants from baseline to 4-week follow-up is shown in Fig. 1.

**Table 3:** Baseline demographic characteristics of the participants in study groups

	Control group (Crest®) (*N* = 30)	Test group (Tiger Herb®) (*N* = 30)
Gender (Count, percent)		
Male	13 (43.3%)	12 (40%)
Female	17 (56.6%)	18 (60%)
Mean age	25.5 ± 1.67	23.5 ± 1.83

Regarding the safety and tolerability of toothpastes, no adverse effects such as allergic reactions were reported in any participants.

Descriptive statistics for baseline logarithm of colony-forming units per milliliter (log CFU/mL) are presented in Table 4. According to the results of the independent samples t-test, there were no significant differences in the baseline salivary colony counts of *Streptococcus mutans* (*P* = 0.811) and *Lactobacillus* (*P* = 0.829) between the experimental groups.

**Table 4:** Mean ± SD ^a^ of baseline and follow-up log CFU/mL ^b^ of salivary microorganisms among the experimental groups

Bacteria	Groups	Baseline		Follow-up	
		Mean ± SD	*P* value^c^	Mean ± SD	*P* value^c^
*Streptococcus mutans*	Crest® (control) (*N* = 30)	7.63 ± 0.57	0.811	7.08 ± 0.57	0.530
*Streptococcus mutans*	Tiger Herb® (test) (*N* = 30)	7.59 ± 0.48		6.98 ± 0.52	
*Lactobacillus*	Crest® (control) (*N* = 30)	7.67 ± 0.62	0.829	7.19 ± 0.52	0.137
*Lactobacillus*	Tiger Herb® (test) (*N* = 30)	7.71 ± 0.62		6.97 ± 0.60	

^a^SD = Standard deviation

^b^Log CFU/mL = logarithm of colony forming units per milliliter

^c^Significance level = 0.05, according to independent samples t-test. (Confidence interval = 95%)

Independent samples t-test also revealed that after four weeks of toothpaste use, no significant differences were observed in the salivary colony counts of *Streptococcus mutans* (*P* = 0.530) and *Lactobacillus* (*P* = 0.137) between the experimental groups (Table 4).

Paired samples t-test revealed that in both groups, salivary colony counts of *Streptococcus mutans* and *Lactobacillus* decreased significantly after four weeks of toothpaste use (*P < 0.001 in Tiger Herb®, and *P = 0.001 in Crest®).

**Fig. 2:**
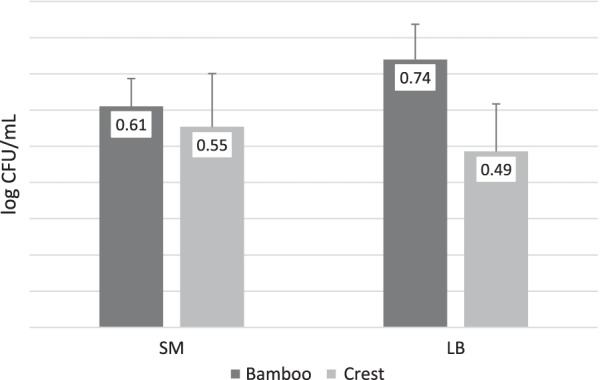
Mean amount of change in the logarithm of colony-forming units per milliliter (log CFU/mL) of *Streptococcus mutans* (SM) and* Lactobacillus* (LB) in case (Tiger Herb®, containing Bamboo salt) and control (Crest®) groups, during the 4-week follow-up period

Furthermore, the mean amount of change was not significantly different between the groups for both *Streptococcus mutans* (*P* = 0.737) and *Lactobacillus* (*P* = 0.126), as shown by the independent samples t-test (Fig. 2).

## Discussion

The primary pathogenic mechanism of dental caries initiation is lactic acid production by dental biofilm bacteria [28]. Acidogenic *Lactobacilli* spp. and *Streptococcus mutans* are the principal pathogens of dental plaque biofilm [29]. Although plaque removal during toothbrushing is mainly accomplished through mechanical action of toothbrushes, the use of appropriate toothpaste can enhance the antibacterial efficacy [30]. The aim of the present randomized controlled clinical trial was to compare the antibacterial efficacy of herbal toothpaste containing Bamboo salt with a non-herbal toothpaste as control, and it was observed that both toothpaste types reduced the *Streptococcus mutans* and *Lactobacillus* counts of saliva equally.

According to previous in-vitro studies, Bamboo salt is proposed to exert a variety of therapeutic effects, making it a potentially suitable ingredient for natural toothpastes [31]. One of them is the ability of Bamboo salt to remineralize incipient enamel lesions [23, 32]. In an in vitro study by Choi et al., a significant improvement in surface hardness of artificial caries-like enamel lesions was observed using the Bamboo salt-sodium fluoride toothpaste [23]. A synergic remineralization potential of Bamboo salt and sodium fluoride was later confirmed in an in vitro study by Kim et al., demonstrating a higher increase in surface hardness by combining both agents, compared to using them separately [32].

Another therapeutic property of Bamboo salt as toothpaste ingredient is its inhibitory effect on cytokines involved in gingival inflammation. In a molecular study on gingival fibroblasts, a reduction in IL-1β, IL-8, TNF-α was observed following the application of Bamboo salt and sodium fluoride, thereby enhancing gingival resistance to inflammation [33]. The anti-gingivitis effect of Bamboo salt was further confirmed in a randomized controlled clinical trial comparing a Bamboo salt toothpaste with another herbal toothpaste containing Centella Asiatica [34].

Regarding the antimicrobial efficacy of Bamboo salt toothpaste, a recent in-vitro study by Lee et al. (2019) investigated the efficacy of Bamboo salt combined with sodium fluoride on *streptococcus mutans*. Various properties attributed to the cariogenesis of this microorganism including its growth, acid production, adherence to glass beads, and expression of *gtf*B, *gtf*C, *gtf*D, and *ftf* genes were significantly restrained by the Bamboo salt and sodium fluoride mixture. It was concluded that Bamboo salt and sodium fluoride can synergically act in inhibiting the cariogenic potential of *Streptococcus mutans* [21], which is consistent with the results of the present in-vivo study.

To the best of our knowledge, no clinical studies have ever investigated the antimicrobial efficacy of Bamboo salt on cariogenic oral bacteria before. Nowadays, dentistry has evolved from a practice-based medical discipline to an evidence-based one. A substantial number of herbal extracts have been incorporated into oral hygiene products such as toothpaste or mouthwash, due to their proposed antimicrobial effects. Although the antimicrobial efficacy of some of them have already been demonstrated in clinical studies, evidence regarding this issue remains limited [35–37].

Patil et al. (2010) compared the anti-microbial efficacy of Himalaya (herbal) and Cheerio gel (fluoridated) toothpaste in an in vivo study and observed both toothpaste types to be effective on salivary *Streptococcus mutans* in children, with no significant difference between them [38]. Pradeep et al. (2012) evaluated the anti-microbial efficacy of aloe vera (herbal) toothpaste in comparison with triclosan (conventional) toothpaste as a placebo in a randomized controlled clinical trial and observed a comparable reduction in microbial counts between them [25].

The present study was similarly designed as a double-blind, parallel, randomized, controlled, clinical trial (RCT). The potential of RCTs to minimize the bias improves their level of evidence, making them the gold standard for the investigation of health care outcomes [39].

One limitation of the present study was attributed to the blinding of participants to the toothpaste type. Although both kinds of toothpaste were mint-flavored and their packages were designed similarly, there was a possibility that the participant detected the taste. Since all the participants were dental students, they might not be representative of the general population, although this could contribute to their improved compliance during the follow-up period. Participants’ compliance could influence the results despite weekly reminder phone calls. Furthermore, the present study only investigated the antibacterial effects and different results might be achieved if other outcomes are investigated; therefore, we suggest that additional outcome measures can be appropriately investigated to highlight the advantages of herbal toothpaste containing Bamboo salt.

The results of this study indicated the promising antibacterial efficacy of toothpaste containing Bamboo salt, which may suggest its administration in self-care oral hygiene procedures. However, further clinical studies on other therapeutic effects of Bamboo salt toothpaste, with a larger sample size, and more extended follow-up period could obtain more definitive conclusions regarding its efficacy for routine daily use.

## Conclusion

Although the herbal toothpaste containing Bamboo salt had no superiority to the routine non-herbal toothpaste type in decreasing oral cavity bacterial load, it can be concluded that it potentially qualifies as a complementary agent for self-care oral hygiene procedures, due to its comparable antibacterial efficacy with the control group.

## Supplementary Information

**Additional file 1**. Raw data: generated and analysed during the study.

## Data Availability

All data generated or analysed during this study are included in this published article [and its supplementary information files].
